# The Mechanisms of the Roles of α-Synuclein, Amyloid-β, and Tau Protein in the Lewy Body Diseases: Pathogenesis, Early Detection, and Therapeutics

**DOI:** 10.3390/ijms241210215

**Published:** 2023-06-17

**Authors:** Moeko Noguchi-Shinohara, Kenjiro Ono

**Affiliations:** Department of Neurology, Kanazawa University Graduate School of Medical Sciences, Kanazawa 920-8640, Japan; m-nohara@med.kanazawa-u.ac.jp

**Keywords:** α-synuclein, amyloid-β, tau protein, aggregation, Lewy body diseases, Parkinson’s disease, dementia with Lewy bodies

## Abstract

Lewy body diseases (LBD) are pathologically defined as the accumulation of Lewy bodies composed of an aggregation of α-synuclein (αSyn). In LBD, not only the sole aggregation of αSyn but also the co-aggregation of amyloidogenic proteins, such as amyloid-β (Aβ) and tau, has been reported. In this review, the pathophysiology of co-aggregation of αSyn, Aβ, and tau protein and the advancement in imaging and fluid biomarkers that can detect αSyn and co-occurring Aβ and/or tau pathologies are discussed. Additionally, the αSyn-targeted disease-modifying therapies in clinical trials are summarized.

## 1. Introduction

The incidence of Parkinson’s disease (PD) has been increasing with the more advanced aging of the population in the world [1]. The prevalence of PD from 1990 to 2015 more than doubled, and it is called “the Parkinson Pandemic” [1]. The biggest risk factor of PD is aging. The clinical manifestations of PD are characterized by bradykinesia, rest tremor, rigidity, and changes in posture and gait. These motor disturbances cause many impairments in activities of daily living. PD is defined as a movement disorder; however, non-motor symptoms, including hyposmia, constipation, urinary dysfunction, orthostatic hypotension, memory loss, depression, pain, and sleep disturbances, are often present in patients with PD, especially in the early stages [2]. The motor signs of PD are linked to nigral degeneration and striatal dopamine depletion, while non-motor symptoms are likely related to neurodegeneration of other structures, including the peripheral autonomic nervous system [3]. Memory loss is one of the non-motor symptoms of PD, and the incidence of dementia in patients with PD is reported to be five times higher than that in healthy older adults. Indeed, dementia was reported to be present in around 50% of 10-year and 90% of 20-year survivors of PD patients [3]. Regarding terminology, dementia with Lewy bodies (DLB) is used when dementia develops before or within one year after the onset of PD. Parkinson’s disease dementia (PDD) is used when dementia occurs more than one year after PD onset [4].

Lewy body dementias include clinically diagnosed DLB and PDD [5], and Lewy body diseases (LBD) are pathologically defined as the accumulation of Lewy bodies in addition to cholinergic deficits [5]. The clinical manifestations of DLB are characterized by dementia as essential features and fluctuating cognition, recurrent visual hallucinations, REM sleep behavior disorder, and Parkinson’s as core clinical features [6]. Additionally, supportive clinical features of DLB are severe sensitivity to antipsychotic agents, postural instability, repeated falls, syncope, or other transient episodes of unresponsiveness, severe autonomic dysfunction, e.g., constipation, orthostatic hypotension, urinary incontinence, hypersomnia, hyposomia, hallucinations in other modalities, systematized delusions, apathy, anxiety, and depression. Regarding Lewy body formation, the aggregation of α-synuclein (αSyn) plays the most important role [7]. In LBD, not only the sole aggregation of αSyn, but also the co-aggregation of amyloidogenic proteins, such as amyloid-β (Aβ) and tau, has been reported [7], and the pathophysiology of co-aggregation of these disease-specific proteins is gathering a lot of attention. Because the aggregation of these disease-specific proteins in the brain begins prior to 20 years of the symptomatic phase of LBD, the development of imaging or fluid biomarkers using disease-specific proteins in cerebrospinal fluid (CSF) and blood is progressing. Additionally, disease-modifying therapies that target αSyn, Aβ, and tau are vigorously developing. In this review, the pathophysiology of co-aggregation of αSyn, Aβ, and tau protein and the advancement in imaging and fluid biomarkers that can detect LBD is discussed. Finally, the αSyn-targeted disease-modifying therapies in clinical trials are summarized.

## 2. Pathophysiology of Co-Aggregation of αSyn, Aβ, and Tau Protein

### 2.1. αSyn in LBD

αSyn is a small (14.5 kDa, 140 amino acids), natively unfolded protein [8]. Historically, αSyn was found in amyloid in neuritic and diffuse plaques of Alzheimer’s disease (AD) brains and named non- Aβ component of AD amyloid [9]. Aggregated αSyn is the major component of Lewy bodies and Lewy neurites. The αSyn’s misfolding and aggregation process represents membrane-bound state monomer converting into oligomers, and oligomers finally convert into highly ordered β-sheet fibrils [10]. Oligomers are defined as the soluble pre-fibrillar intermediate species [10]. A study with post-mortem brains from patients with PD revealed that αSyn oligomer burden was greater in the neocortex, whereas Lewy bodies and Lewy neurites were greater in subcortical regions, including the brainstem [11,12]. When thinking about the pathogenesis of LBD, αSyn oligomers are thought to be the most important state; αSyn oligomers were reported as possibly causing cell death and synaptic dysfunction in vitro [13,14,15] and in animal models [16]. In addition, Sekiya et al. revealed that cognitive impairment was associated with αSyn oligomers in the hippocampus in patients with PD [12].

### 2.2. Cognitive Syndromes of LBD

In LBD, various cognitive domains are affected, such as executive function, memory, and visuospatial function. Cognitive function is significantly related to motor function in LBD patients. In particular, postural instability is significantly associated with visuospatial and executive functions [17]. There are two distinct cognitive syndromes of LBD. Patients with early dysfunction on neuropsychological tests with a posterior cortical basis (memory and visuospatial impairments) progressed more rapidly to dementia [18]. On the other hand, more frontostriatal-based executive dysfunction was not associated with earlier dementia and even improved in some patients [18]. The former is called “posterior cortical” impairments, and the latter is called “frontal executive” impairments. “Posterior cortical” impairments are considered AD comorbidity [19], and “frontal executive” impairments are associated with Lewy body pathology [18,19]. In addition, these cognitive dysfunctions can be observed in the early stages of LBD, and “posterior cortical” and “frontal executive” impairments can be early predictive markers for AD comorbidity and Lewy body pathology, respectively [18].

“Frontal executive” impairments manifest as impairments of executive function, such as deficits in flexibility, planning, and working memory [20]. Impairments of executive function arise from dopaminergic denervation of the striatum. Dopaminergic mediation showed significant effects on executive function in patients with PD in the de novo stage [21]. fMRI study showed that PD patients showed lower planning-related brain activation together with decreased functional connectivity in regions of interest of the bilateral dorsolateral prefrontal cortex, inferior parietal cortex, and caudate nucleus [22].

### 2.3. AD Comorbidity: AD Pathology in LBD

It was reported that up to 50% of LBD patients have AD pathology [7]; the neuropathological study revealed that among the autopsy-confirmed LBD patients (*n* = 213), 56 (26%) had low-level AD pathology, 45 (21%) had intermediate, 63 (30%) had high-level, and 49 (23%) had no AD pathology [23]. In addition, AD comorbidity in LBD is associated with a more severe disease manifestation, cognitive decline, a higher institutionalization risk, and mortality [24]. Co-aggregation of multiple pathogenic proteins is commonly observed in neurodegenerative diseases. Also, αSyn scores, neuritic plaques (aggregation of Aβ), and global cerebral neurofibrillary tangles (aggregation of tau) were all moderately positively correlated with each other [23]. Tau and αSyn have been observed to aggregate together in the same tangles, Lewy bodies, and Lewy neurites in the LBD brain using double immunofluorescence labeling techniques [25].

### 2.4. Cross-Seeding Effects between αSyn, Aβ, and Tau Protein

Regarding the cross-seeding effects between Aβ and αSyn, Aβ was reported to enhance αSyn aggregation in an animal model with neuronal expression of human Aβ and αSyn [26]. The nuclear magnetic resonance spectroscopy study revealed the molecular interaction of αSyn with Aβ [27]. Ono et al. showed that fibrils and oligomers of αSyn, Aβ_1–40_, and Aβ_1–42_ acted as seeds and affected the aggregation of each other in vitro [28]. Regarding the cross-seeding of wild-type αSyn with mutant αSyn seeds, Ono et al. used high-speed atomic force microscopy to determine the kinetics and structural dynamics of αSyn fibril elongation. In that study, Ono et al. found that αSyn sequence variants can produce different types of strains by self- or cross-seeding. Thus, the perpetuation of specific strains would depend on the relative rates of fibril growth and the relative stabilities of the fibrils formed by each strain [29]. Furthermore, Tsigelny et al. reported that αSyn and Aβ could interact directly to form oligomers and proceed to neurodegeneration [30]. However, the mechanism by which AD comorbidity exacerbates the pathogenesis in LBD patients remains unclear. It was reported that the cross-seeding effects of Aβ and αSyn, and αSyn aggregates from LBD patients with comorbid AD pathology were highly toxic to neurons [7,28], particularly the ε4 allele carriers of apolipoprotein E [31]. It was also reported that the cross-seeding effects between αSyn and tau; co-incubation of αSyn and tau synergically promoted aggregation of both proteins in vitro [32]. Moreover, αSyn fibrils had direct cross-seeding effects on tau aggregation, both in neuron cultures and in vivo models [33]. Furthermore, it was noted that cross-seeding effects in αSyn, Aβ, and tau protein occur in two-step processes. First, Aβ plaques induce the pooling of αSyn within the synaptic terminals. Second, Aβ plaques cause the disassembly of microtubules in dystrophic processes. Therefore, these mislocalized αSyn and tau proteins interact with each other. Additionally, it was noted that Aβ plaques enhance αSyn seeding, and then αSyn aggregates induce cross-seeding effects on tau [34]. Further study is needed to understand the cross-seeding process in LBD [7,35].

## 3. Neuroimaging

Neuroimaging tools to detect LBD in patients include head MRI, metabolic PET, dopamine transporter single-photon emission computed tomography (DaT-SPECT), and ^123^I-metaiodovebzylguanidine (MIBG) scintigraphy [5]. Patients with LBD show less medial temporal lobe atrophy than AD on MRI and occipital hypometabolism on metabolic PET [5]. DaT-SPECT can investigate dopamine transporter uptake in basal ganglia, and it also has high sensitivity and specificity for LBD [5]. ^123^I-MIBG scintigraphy also showed excellent sensitivity and specificity in distinguishing patients with LBD from AD; additionally, it is a promising biomarker that reflects postganglionic cardiac sympathetic innervation [5]. Aβ and tau PET have been developed and widely used in diagnosing neurodegenerative diseases; however, the usage of αSyn PET for LBD has never been established [36].

### 3.1. Aβ and Tau PET in LBD

Aβ PET studies have reported that around 50–80% of patients with LBD have AD co-pathology [37,38]. Lee et al. reported that the accumulation of amyloid and tau is greater in primary cortices in LBD patients. Also, the amyloid distribution pattern differs in LBD patients from that of AD. In patients with LBD, amyloid accumulation in primary cortices is greater, whereas that in the temporal cortex is lesser; this suggests that amyloid may play an important role in the tau accumulation in LBD [39].

### 3.2. The Development of an αSyn PET Tracer for LBD

Visualizing αSyn aggregates in the human brain is expected to be useful for early diagnosis of LBD. However, there are no imaging probes that can specifically bind to αSyn aggregates in LBD brain samples; although several imaging probes were reported to strongly bind to αSyn aggregates in vitro [40,41], they had high nonspecific binding and failed to show high binding affinity to LBD brain samples, suggesting unsuitable for assessing αSyn pathology in LBD patients. Furthermore, about half of LBD patients are associated with AD pathology [7]. Thus, both Aβ and αSyn aggregates coexist in some LBD patients, and the levels of αSyn aggregates are lower than that of Aβ aggregates in LBD brains [42]. Therefore, high selectivity probe of αSyn is critically needed to detect αSyn pathology in LBD patients.

Recently, several promising imaging probes which could detect αSyn against Aβ aggregates with high selectivity have been reported. Kaide et al. reported that ^125^I-PHNP-3, which is one of the chalcone analogs with a 4-(dimethylamino) phenyl group, exhibited a high binding affinity for αSyn against Aβ aggregates in human brain samples [42]. The MODAG-001 is another promising lead structure to detect αSyn in LBD patients, which demonstrated high brain uptake and favorable in vivo kinetics and biodistribution [43,44].

## 4. Fluid Biomarkers

The development of pathogenic protein biomarkers in neurodegenerative diseases has recently advanced. In humans, αSyn, Aβ, and tau can be measured not only in CSF markers but also in plasma [45,46,47]. Additionally, salivary αSyn markers have also been developed.

### 4.1. αSyn Biomarker in LBD

Table 1 shows αSyn biomarkers of CSF, blood (plasma/serum), and saliva, skin, and submandibular gland in LBD. CSF αSyn biomarkers in LBD have been largely investigated. Many studies using ELISA revealed that the CSF total αSyn levels are decreased in LBD compared to other neurodegenerative diseases and controls [21,48,49,50,51], whereas the CSF oligomeric αSyn levels are increased in patients with PD compared to controls [46,51], suggesting that CSF total and oligomeric αSyn can be used as diagnostic markers for LBD. Murakami et al. asserted that the CSF total αSyn levels decreased with the deterioration of motor symptoms and cognition in patients with PD [21]. Murakami et al. also showed that the CSF total αSyn levels were positively correlated with CSF Aβ_1–42_ and CSF phosphorylated tau protein (p-tau: phosphorylated at threonine-181), suggesting that both Aβ_1–42_ and p-tau possibly co-aggregated with αSyn in LBD patients [21]. Compta et al. demonstrated that the CSF oligomeric αSyn levels increased in patients with PDD but not in patients with non-demented PD and isolated rapid-eye-movement sleep behavior disorder (iRBD) [52], which was considered prodromal stages of α-synucleinopathies [53]. The CSF phosphorylated αSyn concentrations were higher in patients with PD compared to the control group [54].

The real-time quaking-induced conversion (RT-QuIC) assay platform for ultrasensitive detection of αSyn was developed, and the validation study using CSF samples of neuropathologically confirmed DLB and PD patients revealed high sensitivity (98%) and specificity (100%) [55]. RT-QuIC can also detect misfold αSyn in CSF in patients with iRBD, and the longitudinal study showed that αSyn positivity in patients with iRBD was associated with an increased risk of subsequent LBD diagnosis [56]. Therefore, detecting misfold αSyn in CSF represents a potential prodromal marker of LBD [56]. The αSyn seed amplification assay (SAA) performed on CSF distinguish patients with PD from healthy controls with high sensitivity and specificity, but results vary depending on the presence of the LRRK2 Gly2019Ser variant, as well as clinical features, particularly hyposmia [57].

Regarding the blood αSyn markers, the meta-analysis indicated that total αSyn levels in the blood (plasma and/or serum) significantly increased in PD patients compared to controls [58]. Several studies reported that phosphorylated αSyn levels in plasma significantly increased in PD patients compared to controls [59,60], and Chatterjee et al. reported that serum levels of phosphorylated αSyn significantly elevated in later stages of PD [61]. Whereas a few studies reported that the levels of oligomeric αSyn in blood in patients with LBD and the results were controversial; one study showed that the oligomeric αSyn levels in the blood significantly elevated in patients with PD [62], while the other found no differences between the groups [63]. Lewy body pathology was found in the submandibular gland [64] and skin [65]. Some studies found that the saliva αSyn levels could differentiate LBD from controls [66,67]. RT-QuIC and protein misfolding cyclic amplification (PMCA) assays for the detection of αSyn using skin samples were developed for skin biomarkers for diagnosis of PD [68,69]. Studies revealed that both RT-QuIC and PMCA assays using autopsy abdominal skin samples from PD cadavers or posterior cervical and leg skin biopsy tissues from living PD patients showed high sensitivity and specificity [68,69].

The Systemic Synuclein Sampling Study (S4) measured αSyn in biopsies of skin, colon, submandibular gland, CSF, saliva, and plasma. S4 revealed that levels of CSF total αSyn were lower in PD patients compared to controls, but specificity was low; αSyn immunoactivity in the skin and the submandibular gland was specific for PD, but sensitivity was low [70]. BioFIND investigated the relationships of CSF αSyn, plasma αSyn, and saliva αSyn in patients with PD [71]. Although the plasma and saliva αSyn levels neither showed differences between the groups nor correlated with the CSF αSyn levels, they found that the CSF αSyn levels were lower in PD versus controls [71].

**Table 1:** α-Synuclein biomarkers of CSF, blood, and saliva in Lewy body diseases.

Study	Marker	Type of Specimen	LBD Subgroup	*n* (LBD)	Autopsy/ Clinically Diagnosed	*n* (Control)	Results	AUC
Mollenhauer 2011 [50]	total αSyn	CSF	DLB	13	autopsy cases	21 (AD)	Decreased total αSyn level in LBD	0.687
Mollenhauer 2011 [50]	total αSyn	CSF	PD, DLB	314	clinically diagnosed	46 (NPH, PSP)	Decreased total αSyn level in LBD	0.711
Shi 2011 [48]	total αSyn	CSF	PD	126	clinically diagnosed	137 (normal)	Decreased total αSyn level in LBD	0.71
Parnetti 2014 [51]	total αSyn	CSF	PD	44	clinically diagnosed	25 (normal)	Decreased total αSyn level in LBD	0.68
Goldman 2018 [71]	total αSyn	CSF	PD	115	clinically diagnosed	88 (normal)	Decreased total αSyn level in LBD	-
Chahine 2020 [70]	total αSyn	CSF	PD	59	clinically diagnosed	21 (normal)	Decreased total αSyn level in LBD	-
Tokuda 2010 [46]	oligomeric αSyn	CSF	PD	32	clinically diagnosed	28 (normal)	Increased αSyn oligomers level in LBD	0.859
Parnetti 2014 [51]	oligomeric αSyn	CSF	PD	44	clinically diagnosed	25 (normal)	Increased αSyn oligomers level in LBD	0.72
Compta 2015 [52]	oligomeric αSyn	CSF	PDD	20	clinically diagnosed	13 (normal), 23 (iRBD)	Increased αSyn oligomers level in LBD	-
Wang 2012 [54]	phosphorylated αSyn	CSF	PD	93	clinically diagnosed	78 (normal)	Increased phosphorylated αSyn level in LBD	-
Iranzo 2013 [53]	αSyn (RT-QuIC)	CSF	IRBD	44	clinically diagnosed	-	Detected αSyn in IRBD	-
Bargar 2021 [55]	αSyn (RT-QuIC)	CSF	PD, DLB	146	autopsy cases	23 (normal)	Detected αSyn in LBD	-
Siderowf 2023 [57]	αSyn (SAA)	CSF	PD	545	clinically diagnosed	163 (normal)	Positive in LBD	-
Zubelzu 2022 [58]	total αSyn	plasma/serum	PD	2683	clinically diagnosed	1838 (normal)	Increased total αSyn level in LBD	-
Goldman 2018 [71]	total αSyn	plasma	PD	115	clinically diagnosed	88 (normal)	no significant changes	-
Foulds 2013 [59]	phosphorylated αSyn	plasma	PD	189	clinically diagnosed	91 (normal)	Increased phosphorylated αSyn level in LBD	0.717
Lin 2019 [60]	phosphorylated αSyn	plasma	PD	122	clinically diagnosed	68 (normal)	Increased phosphorylated αSyn level in LBD	0.94
Wang 2020 [62]	phosphorylated αSyn	plasma	PD	40	clinically diagnosed	40 (normal)	Increased phosphorylated αSyn level in LBD	-
El-Agnaf 2006 [72]	oligomeric αSyn	plasma	PD	34	clinically diagnosed	27 (normal)	Detected αSyn in LBD	-
Wang 2020 [62]	oligomeric αSyn	plasma	PD	40	clinically diagnosed	40 (normal)	Increased αSyn oligomers level in LBD	-
Emelyanov 2017 [63]	oligomeric αSyn	plasma	PD	17	clinically diagnosed	18 (normal)	no significant changes	-
Al-Nimer 2014 [66]	total αSyn	saliva	PD	20	clinically diagnosed	20 (normal)	Decreased total αSyn level in LBD	-
Goldman 2018 [71]	total αSyn	saliva	PD	115	clinically diagnosed	88 (normal)	no significant changes	-
Kang 2016 [67]	oligomeric αSyn	saliva	PD	201	clinically diagnosed	67 (normal)	Increased αSyn oligomers level in LBD	-
Chahine 2020 [70]	αSyn (Immunoreactivity)	skin	PD	59	clinically diagnosed	21 (normal)	Detected αSyn in LBD	-
Manne 2020 [69]	αSyn (RT-QuIC)	skin	PD	25	clinically diagnosed	25 (normal)	Detected αSyn in LBD	-
Wang 2021 [68]	αSyn (RT-QuIC)	skin	PD	47	autopsy sample	43 (nonneurodegenerative controls)	Detected αSyn in LBD	0.99
Wang 2021 [68]	αSyn (PMCA)	skin	PD	47	autopsy sample	43 (nonneurodegenerative controls)	Detected αSyn in LBD	-
Wang 2021 [68]	αSyn (RT-QuIC)	skin	PD	20	clinically diagnosed	21 (normal)	Detected αSyn in LBD	0.99
Wang 2021 [68]	αSyn (PMCA)	skin	PD	10	clinically diagnosed	10 (normal)	Detected αSyn in LBD	0.92
Chahine 2020 [70]	αSyn (Immunoreactivity)	submandibular gland	PD	59	clinically diagnosed	21 (normal)	Detected αSyn in LBD	-

The AUC value showed the diagnostic ability to differentiate LBD from controls.. Abbreviations: AD, Alzheimer’s disease; αSyn, α-Synuclein; AUC, area under the curve; CSF, cerebrospinal fluid; DLB, dementia with Lewy bodies; iRBD, isolated rapid-eye-movement sleep behavior disorder; LBD, Lewy body diseases; NPH, normal pressure hydro-cephalus; PD, Parkinson’s disease; PSP, progressive supranuclear palsy; RT-QuIC, real-time quaking-induced conversion.

### 4.2. Aβ Biomarker in LBD

It was reported that 40% of patients with LBD have a CSF profile compatible with AD [73], i.e., CSF Aβ reduction and CSF tau elevation. The lower CSF Aβ_1–42_ levels were reported to be a predictive factor of cognitive decline in LBD [74,75]. Steenoven et al. reported different profiles of CSF Aβ reduction in LBD and AD; LBD showed a significant reduction of CSF Aβ_1–38_, Aβ_1–40_, and Aβ_1–42_, whereas AD was characterized only by lower CSF Aβ_1–42_ levels [76]. The mechanisms underlying the differences in CSF Aβ profiles between LBD and AD remain unknown, although a possible dysregulation in APP pathways could be a viable explanation [76]. Further research is needed to clarify the pathophysiological mechanisms underlying α-Syn and Aβ aggregation and toxicity in LBD patients.

Reflecting the comorbidity with AD, the levels of blood Aβ biomarkers in LBD were observed to have high variabilities. The large study using the Quanterix Simoa Human Neurology 4-Plex E assay showed that plasma Aβ_1–42_/Aβ_1–40_ had a limited ability to classify AD from LBD [77]. Additionally, the same study described that there were no significant differences in the plasma Aβ_1–42_/Aβ_1–40_ levels between PET-Aβ-positive (*n* = 29) and PET-Aβ-negative LBD (*n* = 30) [77]. Recently, the plasma Aβ biomarkers in Aβ-positive (*n* = 10) and Aβ-negative LBD patients (*n* = 15) using immunoprecipitation–mass spectrometry (IP–MS) assay-based techniques were investigated, and it was found that the plasma Aβ_1–42_/Aβ_1–40_ ratio was significantly decreased in patients with Aβ-positive LBD compared to those with Aβ-negative LBD, suggesting plasma Aβ_1–42_/Aβ_1–40_ ratio measured by IP–MS assay might be a useful marker for comorbid AD pathology in LBD [78].

### 4.3. Tau Biomarker in LBD

The CSF total tau protein levels (t-tau) significantly increased in patients with dementia, including AD, DLB, and PDD, compared to individuals with normal cognition [79]. The CSF levels of t-tau and p-tau in patients with DLB were higher compared to patients with PD and PDD [79,80]. When the association between the levels of CSF biomarkers and AD co-pathology in LBD was examined, the medium/high AD co-pathology group showed significantly higher CSF t-tau levels and lower Aβ_1–42_ levels compared to the low/no AD co-pathology group; however, CSF p-tau levels did not differ between with and without AD co-pathology in patients with LBD [81].

Regarding plasma tau biomarkers, it was reported that the levels of plasma phospho-tau 217 and phospho-tau 181 were correlated with tau PET and CSF Aβ_1–42_/Aβ_1–40_ ratio in LBD [82], suggesting plasma phospho-tau may be useful to detect AD co-pathology in patients with LBD.

## 5. Disease-Modifying Therapy

As mentioned above, aggregated αSyn is considered a main pathological feature of LBD, and immunotherapy targeting extracellular αSyn has been proposed for disease-modifying therapy. Disease-modifying therapy or curative treatment is not available for LBD; therefore, development in immunotherapy targeting αSyn is progressing. There are several disease-modifying approaches for aggregated αSyn, and potential therapeutic compounds, including αSyn monoclonal antibodies, αSyn peptide vaccine, nucleotide medicines, epigenetic therapies, and αSyn misfolding inhibitors targeting αSyn, have been proposed. Table 2 shows αSyn-targeted disease-modifying therapies in clinical trials.

### 5.1. Immunotherapies Targeting αSyn

Several active and passive immunotherapies targeting αSyn have been investigated. Prasinezumab is a monoclonal antibody that selectively binds aggregated αSyn at the C-terminal of the protein, and it reduces the accumulation of intraneuronal αSyn aggregates and improves functional performance in water-maze and horizontal-beam test in animal models of α-synucleinopathy [83,84,85]. In phase 1 trials, prasinezumab showed brain penetration and reduced serum αSyn levels in humans [86,87]. Recently, the results of the Phase 2 Trial of Anti α-Synuclein Antibody in Early Parkinson’s Disease (PASADENA) study using prasinezumab were reported [88]. The PASADENA study was composed of three parts: a 52-week, double-blind, placebo-controlled part (part 1), an exploratory additional 52-week blinded extension (part 2), and a 5-year open-label extension (part 3). Participants with early PD are to receive an intravenous placebo or prasinezumab at a dose of 1500 mg or 4500 mg every 4 weeks for 52 weeks. The primary endpoint was the change from baseline to week 52 in the sum of scores on parts I, II, and III of the Movement Disorder Society-sponsored revision of the Unified Parkinson’s Disease Rating Scale (MDS-UPDRS). A total of 316 participants were enrolled in the PASADENA study. The baseline mean MDS-UPDRS scores were 32.0 in the placebo, 31.5 in the 1500 mg, and 30.8 in the 4500 mg group. Mean changes from baseline to 52 weeks were 9.4 in the placebo, 7.4 in the 1500 mg, and 8.8 in the 4500 mg group, resulting in no significant differences between the groups. There was also no substantial difference between the group’s dopamine trans-porter levels on DaT-SPECT [88] (Table 2). Cinpanemab is a monoclonal antibody that selectively binds αSyn oligomers/fibril at the N-terminal of the protein. SPARK study is a 52-week, multicenter, double-blind, phase 2 trial using cinpanemab. Participants with early PD were to receive intravenous infusions of placebo (control) or cinpanemab at a dose of 250 mg, 1250 mg, or 3500 mg every 4 weeks, followed by an active-treatment dose-blinded extension period for up to 112 weeks. A total of 357 participants were enrolled. The baseline mean MDS-UPDRS scores were 31.9 in the placebo, 31.9 in the 250 mg, 32.9 in the 1250 mg, and 32.6 in the 3500 mg group. Mean changes from baseline to 52 weeks were 10.8 in the placebo, 10.5 in the 250 mg, 11.3 in the 1250 mg, and 10.9 in the 3500 mg group, resulting in no significant differences between the groups. DaT-SPECT imaging at 52 weeks showed no differences between the control group and any cinpanemab group [89] (Table 2). The PASADENA and SPARK studies have similar trial designs, and both trials used the change in the sum of scores on parts I, II, and III of the MDS-UPDRS as a primary endpoint. Similar numbers of participants with early PD were enrolled (316 in PASADENA and 357 in the SPARK study), as well as DaT-SPECT included in the secondary endpoints to assess the rate of decline in dopamine terminal integrity [88,89]. Both PASADENA and SPARK studies showed no change with regard to DaT-SPECT [88,89]. Across the striatal binding ratios of the striatum, putamen, and caudate, DaT-SPECT imaging showed no substantial differences between the control (placebo) group and any prasinezumab and cinpanemab group [88,89]. These failures of the clinical trials using anti-αSyn monoclonal antibodies seem to be the end of the road for monoclonal antibodies in the treatment of early PD [90]. However, the timing of therapeutic intervention in PD may be a factor in the failures of agents targeted to a misfolded αSyn protein. In patients with PD, the entrance of αSyn oligomers into cells may be an early event that progresses to cellular dysfunction. Therefore, treatments with prasinezumab and/or cinpanemab in preclinical or prodromal stages of PD may be valuable [90].

Monoclonal antibodies that selectively bind aggregated αSyn at the C-terminal, namely MEDI1341 and Lu AF82422, are investigated in phase 1 trials [91] (Table 2).

### 5.2. Nucleotide Medicines and Epigenetic Therapies Targeting αSyn

Reducing synuclein alpha (*SNCA*) expression levels may delay the disease course because the *SNCA* gene multiplication causes familial PD, namely autosomal dominant Parkinson’s disease-4 (PARK4). Several antisense oligonucleotides (ASO) have been developed [92]; an amido-bridged nucleic acid (AmNA)-ASO, one of the ASOs, that targeted *SNCA* successfully downregulated *SNCA* at both the mRNA and protein levels in vitro and in vivo [92]. Additionally, AmNA-ASO was efficiently delivered into the mouse brain by intracerebroventricular injection and ameliorated neurological defects in the PD mouse model expressing human wild-type *SNCA* [92]. Phosphen is an epigenetic drug that is also known as butanetap. It reduced α-synuclein expression in the brain and gut and improved intestinal function in the A53T α-synuclein transgenic mouse model of PD [93]. Annovis Bio reported significant reductions in inflammatory markers, namely sTREM2 and GFAP, in butanetap-treated PD patients [94]. The phase 3 trial has started evaluating the efficacy of butanetap [91] (Table 2).

### 5.3. Inhibition of αSyn Aggregation, Enhancement of αSyn Clearance, and Other Strategies

UCB0599 is a small-molecule αSyn aggregation inhibitor. It interacts with the C-terminal domain of αSyn and is reported to reduce retinal αSyn pathology in mice expressing human αSyn. UCB0599 also reduced cortical αSyn pathology, astrogliosis, normalized striatal dopamine transporter levels, and improved motor function [95]. Anle138b is another αSyn aggregation inhibitor, which was effective in mouse models of αSyn and tau pathology. It decreased neuron loss, increased survival, and improved movement [96,97]. MODAG has investigated a Phase 1b study in 48 people with PD to assess the safety, tolerability, and pharmacokinetics of 150 mg anle138b [91] (Table 2). NPT520-34 is a small-molecule toll-like receptor (TLR2) antagonist, which recognizes aggregated proteins, and downregulates autophagy. Based on the information available on the company’s website, NPT520-34 attenuates neuroinflammation mediated by microglia and astrocytes and reduces neuropathic protein levels, including αSyn. Trehalose showed a reduction in the accumulation of N-ethylmaleimide sensitive factor deposits in neurons, characterized for LRRK2 mutation, in a mouse model. Additionally, trehalose also showed a significant improvement in motor and cognitive performance in the mouse model. Phase 4 study aiming to evaluate the safety and tolerability of trehalose in idiopathic PD and PD carrying the LRRK2 mutation is ongoing [91] (Table 2).

In the PD animal model, Abelson tyrosine kinase (c-Abl) activation is essential for initiating and progressing αSyn pathology. The c-Abl inhibitor lkT-148009 suppressed c-Abl activation and protected dopaminergic neurons from degeneration in a mouse model of PD. PD mice treated with lkT-148009 showed a significant reduction in αSyn pathology and better motor function [98]. The phase 2 trial to evaluate a clinical benefit in patients with PD has begun [91] (Table 2).

**Table 2:** α-Synuclein-targeted disease-modifying therapies for Parkinson’s disease in clinical trials.

Drug	Phase	*n*	Duration	Primary Outcome Measures	Results	NCT Number	Study Name	Reference
Prasinezumab	2	316	52 weeks	UPDRS part I, II, and III	not significance	NCT03100149	PASADENA study	[88]
Cinpanemab	2	357	52 weeks	UPDRS part I, II, and III	not significance	NCT03318523	SPARK study	[89]
MEDI1341	1	25	28 weeks	Safety, tolerability, pharmacokinetics, and pharmacodynamics	ND	NCT04449484	n.a	[91]
Lu AF82422	1	74	84 days	Safety and tolerability	ND	NCT03611569	n.a	[91]
UB-312	1	138	44 weeks	Safety, tolerability, and immunogenicity	ND	NCT04075318	n.a	[91]
Butanetap	3	450	6 months	UPDRS parts II and III, safety and tolerability	ND	NCT05357989	n.a	[93]
Anle138b	1	70	6 weeks	Safety, tolerability, pharmacokinetics, and pharmacodynamics	ND	NCT04685265	n.a	[91]
Trehalose	4	20	36 weeks	Safety and efficacy	ND	NCT05355064	n.a	[91]
lkT-148009	2	120	12 weeks	Safety, tolerability, and pharmacokinetics	ND	NCT05424276	n.a	[98]

Abbreviations: n.a, no applicable; NCT number, national clinical trial number; ND, no data; UPDRS, unified Parkinson’s disease rating scale.

Ono et al. showed that phenolic compounds such as the wine-related polyphenol myricetin (Myr), a major component of curry spice turmeric curcumin (Cur), rosmarinic acid (RA), nordihydroguaiaretic acid (NDGA), and ferulic acid (FA), inhibited the formation of αSyn fibrils, as well as destabilized preformed fibrils [99]. Moreover, Ono et al. revealed that the phenolic compounds Myr, FA, NDGA, Cur, and RA had inhibitory effects on αSyn oligomerization using the photo-induced cross-linking of unmodified proteins studies. Ono et al. also revealed the ability of Myr to inhibit αSyn oligomerization and secondary structure conversion. Additionally, Myr is directly bound to the first nine residues of the N-terminus of αSyn. Electrophysiological assays for long-term potentiation in mouse hippocampal slices revealed that Myr ameliorated αSyn synaptic toxicity by inhibition of αSyn oligomerization [100]. These results showed that Myr prevents the αSyn aggregation process and reduces the neurotoxicity of αSyn oligomers, suggesting that phenolic compounds, including Myr, would be considerable candidates for disease-modifying therapies for α-synucleinopathies. Ono also showed that phenolic compounds such as Myr, Cur, RA, NDGA, and FA inhibit the formation of Aβ fibrils as well as dissociate preformed fibrils [101,102]. Especially, RA can inhibit the aggregation, including oligomerization of Aβ, resulting in the decrease of cyto- and synaptic toxicities [102]. We conducted the randomized placebo-controlled double-blind trials aimed to assess (i) pharmacokinetics [103], (ii) safety and tolerability [104], and (iii) efficacy of lemon balm (*Melissa officinalis*: *M. officinalis*) extract containing RA on cognition in older adults without dementia [105]. We conducted three randomized placebo-controlled double-blind trials; the first one was performed in healthy individuals (*n* = 11) to assess the tolerability and safety of *M. officinalis* extract capsule, and the second one aimed to show the safety, tolerability, and efficacy in patients with mild AD dementia (*n* = 20), and the third one investigated the effects on cognition in older adults (*n* = 323) without dementia. The results indicate that *M. officinalis* extract is tolerable and safe in healthy individuals and patients with mild AD dementia. Additionally, *M. officinalis* may prevent cognitive decline in older adults without hypertension. Regarding tau protein, epicatechin, catechin, and epigallocatechin-3-gallate, which are richly contained in green tea, oolong tea, and black tea, are believed to prevent the aggregation of tau protein [106,107].

## 6. Discussion and Conclusions

In LBD patients, AD comorbidity is very common, and the co-aggregation of multiple pathogenic proteins, such as αSyn, Aβ, and tau, is frequently observed. Therefore, the presence of AD comorbidity in LBD patients is important for their clinical management, particularly if new disease-modifying therapies targeting αSyn, Aβ, and tau pathologies have been developed. Aβ and tau have been thought to play an important role in AD pathogenesis. However, the repeated failures of clinical trials on vaccines and humanized anti-Aβ and anti-tau monoclonal antibodies have resulted in doubts about this strategy. More recently, two new anti-Aβ monoclonal antibodies (Aducanumab and Lecanemab) have been approved by the US Food and Drug Administration; the approvals of those human monoclonal Aβ antibodies have brought new hope to patients of AD and shed new light on the research and innovation to conquer the disease. Aducanumab is a human IgG1 monoclonal antibody that binds to the N terminus of Aβ in an extended conformation [108]. Lecanemab is humanized IgG1 monoclonal antibody preferentially targeting soluble aggregated Aβ and possessing activity across oligomers, protofibrils, and insoluble fibrils [108]. We recently used high-speed atomic force microscopy to observe the structural dynamics of Aβ_1–42_ protofibrils at the single-molecule level and the effect of Lecanemab. We found that Lecanemab remained stable in binding to protofibrils and to globular oligomers, inhibiting the formation of large aggregates. These results provide direct evidence for a mechanism by which antibody drugs interfere with the Aβ aggregation process [109]. Another major hallmark of AD is abnormally phosphorylated tau protein. Tau protein appears to be better correlated with the severity of cognitive decline than Aβ in AD patients. Several anti-tau immunotherapies are in clinical trials. When considering disease-modifying therapies in patients with LBD, the issue of co-occurring pathologies other than αSyn differing in each patient might occur. Therefore, it is important to develop imaging and fluid biomarkers to assess the status of comorbid pathologies.

In this review, the pathophysiology of co-aggregation of αSyn, Aβ, and tau protein was described. Additionally, the imaging and fluid biomarkers of LBD, including MRI, SPECT, CSF, blood, and saliva, were summarized. The development of the αSyn PET tracer is expected to evaluate the disease progression. Also, ongoing αSyn-targeted disease-modifying therapies that are under development were summarized. Comorbid pathologies and other factors could cause variations in the symptoms experienced by patients. Hence, LBD is considered to be a heterogeneous disease. In the future, patient-personalized treatment regimens developed by comorbid pathologies may lead to better treatments.

## Data Availability

Data sharing is not applicable to this article.
